# Equifinality and the Key Role it Plays in Understanding the Future of Leadership

**DOI:** 10.58315/jcld.v10.282

**Published:** 2023-10-27

**Authors:** Sam Hunter, Austin C. Doctor, Matthew Allen, Gina S. Ligon

**Affiliations:** National Counterterrorism Innovation, Technology, and Education (NCITE) Center of Excellence, University of Nebraska Omaha; National Counterterrorism Innovation, Technology, and Education (NCITE) Center of Excellence, University of Nebraska Omaha; National Counterterrorism Innovation, Technology, and Education (NCITE) Center of Excellence, University of Nebraska Omaha; National Counterterrorism Innovation, Technology, and Education (NCITE) Center of Excellence, University of Nebraska Omaha

**Keywords:** Equifinality, Pathways, Leadership, CIP, Diversity

## Abstract

As organizations continue to increase in diversity across a range of demographic, value-based, and attitudinal variables, there can be a natural tension around differing styles and approaches to leading. We offer that these differences need not necessarily serve as a source of conflict if organizations are able to embrace the principle of equifinality. Equifinality, applied to leadership, represents the notion that there is more than one pathway to leading successfully. By focusing on equifinality as a core principle, stylistic differences can add to the fabric of organizational life rather than being a source of tension in it. We offer an example of an equifinality based approach to leading, the charismatic, ideological, and pragmatic (CIP) model to illustrate equifinality successfully applied as a core principle. We conclude by offering practical guidance on how to effectively apply an equifinality approach to leadership in organizations.

As researchers at the National Counterterrorism Innovation, Technology, and Education Center of Excellence, we engage with scholars and practitioners across the national, and increasingly international, homeland security enterprise (HSE). A pleasant observation made with ongoing engagement in the HSE is how varied and unique the community is. That is, there is notable diversity in the HSE, defined as those with a vested interest in security across all levels of government, nonprofits (including academia), private sector, and community members. We use diversity here as a broad term to non-exhaustively include demographic characteristics such as age, race, ethnicity, country of origin, sexual orientation, gender, and gender identification, as well as more readily perceived positional forms such as academic discipline (e.g., psychology, political science, criminology, and management), agency or department (e.g., DoD, DHS, FBI, CIA, and Home Office), and attitudinal forms of diversity such as political affiliation.

The diversity across the HSE is not entirely surprising given demographic shifts in the U.S. where the most recent Census reveals significant increases in racial and ethnic diversity, as well as within National Security elements such as the Department of Homeland Security whose workforce diversity is “almost double the federal workforce benchmarks” (DHS Inclusive Strategic Plan, 2022, p. 6). We are becoming more diverse as a nation, and our security apparatus appropriately, if imperfectly, increasingly reflects that.

Organizational diversity that accurately reflects the broader national population is a noble if not wholly necessary goal. Yet, given current and recent tensions within the U.S. and across the globe, such an ethical and even pragmatic aim does not come without a cost. Differences, be they actual or perceived, are often a source of conflict and tension within organizations (e.g., Jehn et al., 2008). As extremism and terrorism researchers, we are all too familiar with the extreme ends of this tension, studying groups that seek to accelerate race wars, observing individuals with increased hatred and potential for violence against elected officials, and researching the ideology of groups who seek to attack or even overthrow the U.S. government.

Tension surrounding differences is not limited to hiring and promotion initiatives or national demographic shifts and has extended to the study of leadership as well. Leadership approaches more frequently adopted by women versus men (and vice-versa), for example, are pitted against one another with researchers offering that one approach is superior to the other (e.g., Rosette & Tost, 2010). Early research on the topic of implicit leadership revealed that for many subordinates, if a leader physically looked a certain way (e.g., tall, white, and male), their behaviors were seen as more competent than those who did not fit that stereotypic mold. The extension of such an observation was taken by some to indicate that there was a singular ideal leader type. As a counterpoint, research on women in leadership roles exalted that in the modern era of work, communal and relationship-oriented qualities stereotypically linked to female leaders were superior to those qualities more frequently associated with male leaders—a phenomena termed the “female leadership advantage” (Eagly & Carli, 2003; Post et al., 2019). Generational differences are also discussed with seemingly greater frequency, with some suggesting traditional forms of leadership are outdated, and younger followers need alternative “fresh” forms of leadership. Older generations, some suggest, pine for a time when leaders operated in a way they most strongly identified with (Salahuddin, 2010).

Debates over differences in leaders and leadership also extend beyond the surface level (i.e., demographic differences) to differences in leaders’ methods of influence. One of the most pervasive of these is between transformational or transactional models of leadership (Bass, 1990), with several scholars offering that transformational leadership being superior and transactional being inferior. Stated differently, a natural by-product of such framing is the pervasive belief there is one best way to lead, and that alternatives are simply inferior if not harmful to leader and organizational performance.

Building off more than 25 studies across 20 years (Hunter & Lovelace, 2020, 2022; Lovelace et al., 2019), we challenge this implicit view and offer that such a singular approach is flawed in its underlying premise and will perpetually result in unnecessary, counterproductive conflict. Instead, we argue that a simple idea affords a more tenable and sustainable path forward. Namely, leadership researchers and practitioners would benefit from embracing the principle of equifinality.

The premise of equifinality—*that there is more than one path to reach the same outcome*—has early roots in the fields of biology and physics (Von Bertalanffy, 1950). Outside of leadership, there are several illustrations of equifinality. Yet within the field, these principles have not been widely embraced. This is not to say that all leadership researchers have avoided the topic. Indeed, a few have tried. Hackman and Wageman (2007), for example, suggest that equifinality would be quite useful in the study of leadership, but noted that the core notion of multiple pathways is often missed due to a preference for static, singular, or fixed approaches. This is well-illustrated by the fact that leadership research has been dominated by a few frameworks in recent years, most notably transformational leadership. In the management field, Ashmos and Huber (1987) as well as Gresov and Drazin (1997) lamented that equifinality as a concept was not more prominent, noting it as one of the key “missed opportunities” (Gresov & Drazin, 1997, p. 404) in the study of systems and management.

In the vein of the researchers above, there have been a few rare examples of successfully applying equifinality to understanding leaders and managers. In the organizational strategy literature, Porter (1980) offers that a competitive edge could be gained via three equally viable strategic approaches: being unique and different with a focus on change; being focused on what was done previously; and pragmatically tackling cost issues. Relatedly, Miles and colleagues suggest that organizations could manage change using differing yet equally viable tactics that included: *prospectors* who emphasize change (Miles et al., 1978), *defenders* who sought stability via insulation and a narrowed focus, and *analyzers* who keep an eye on emerging trends, shifting to engage in problem-solving as needed. Perhaps most impactfully, in their work on systems theory, Katz and Kahn offer that equifinality happens when “a system can reach the same final state, from different initial conditions and by a variety of different paths” (1978, p. 30).

Although less popular in the study of leadership, multiple viable pathways to achievement have been observed in research areas outside of leadership. This includes the education literature, where mastery pathways and performance-approach pathways both led to success (Harackiewicz & Linnenbrink, 2005). Other areas include engagement in collective action (e.g., Saab et al., 2015), where researchers found that some individuals chose to engage in collective action due to strongly identifying with a root cause, with others engaging in collective action due to a more rational cost-benefit approach. In the area of innovation, creativity researchers have hypothesized that individuals with approach-oriented traits and avoidance-oriented traits are both capable of creative performance, yet the differing orientations result in different pathways to achievement.

Finally, Weber (1924, 1947) suggested that for managers, there were three primary forms of authority. Rational authority, he argued, derives from the perceived competence of a leader, resulting in follower stability, clarity, and perhaps most critically, efficiency on the part of the follower. Traditional authority was derived from an emphasis on core values and traditions, with followers sharing those values being most impacted by that form of authority. Weber described charismatic authority as the least common type of authority, occurring where followers believe the leader possessed special qualities and are drawn to the rarity and positive appeal of their charisma. Mumford (2006) and more recently some of our own work (e.g., Hunter & Lovelace, 2020; Ligon et al., 2020) returned to the original work of Weber (1924) and proposed that there were three viable pathways to outstanding leadership: charismatic, ideological, and pragmatic (CIP).

## CIP Theory of Leadership: An Example of a Successful Equifinality-Based Approach

Although there are number of frameworks that may be showcases as illustrations of equifinality, few have the concept as foundational to their theories. As such, we offer CIP as a non-exhaustive illustration of how equifinality can successfully be used as a foundation in thinking about leadership. The CIP theory is grounded in the notion of providing sensemaking to followers, drawing on the leader’s view of the world. Specifically, leaders are theorized to experience life events that shape how they believe the world operates, and those beliefs, in turn, shape how the leader makes sense of the world when engaging with followers. Differing, meaningful life events, therefore, represent a key driving force in shaping CIP forms of leadership (Ligon et al., 2008).

### Charismatic Pathway

Charismatic leaders inspire others to act via a compelling, positive, future-oriented vision. They make broad appeals to a wide range of individuals, offering a sentiment of hope to produce positive outcomes for everyone. Exemplar charismatic leaders have included: Lee Iocca, Eva Peron, David Ben-Gurion, Henry Ford, Franklin Roosevelt, John F. Kennedy, Pete Carrol, and Margaret Thatcher.

### Ideological Pathway

Ideological leaders have a narrower appeal as compared to charismatic leaders, yet this appeal is often quite powerful as it is based on a shared belief system. Ideological leaders offer that the best path forward is deeply grounded in tradition and a return to behavior normative of a previous era, when the values they embody were perceived to be most represented. These leaders are more likely to utilize negative affect to compel followers to recreate a period where such values can thrive once again. Exemplar ideological leaders have included: Betty Friedan, Emma Goldman, Ronald Reagan, Paul “Bear” Bryant, Jane Addams, W.E.B. du Bois, Lech Walesa, and Mohandas Ghandi.

### Pragmatic Pathway

As compared to charismatic leaders and even ideological leaders, pragmatic leaders are least likely to fit a stereotypical view of leadership, relying less often on emotion or inspiration and instead acting as rational problem solvers. Pragmatic leaders are focused on finding solutions, using logic rather than emotion to engage with their followers. Exemplar pragmatic leaders have included: Walt Disney, Katharine Graham, Thomas Watson Alfred Dupont, Mikail Gorbechev, Sam Walton, Bill Belichick, and Warren Buffet.

## Empirical Support and Key Findings of CIP Leadership Theory

Perhaps the most compelling result of the nearly 20 years and more than 25 investigations of the CIP theory is the consistent *non-finding* of performance differences across successful leaders (see Allen et al., 2020 for review). Whether the method be a historiometric, content analysis of world leaders (Mumford, 2006), college and NFL football coaches (Hunter et al., 2011), a lab-based computer simulation (Hunter et al., 2009), or a case-analysis of civil rights leader exchanges (Bedell-Avers et al., 2008), results have consistently revealed that all three pathways offer viable routes to success and impact. This is not to say there has been a complete dearth of differences in outcomes when moderators such as nature of the task or phase of a project were taken into account. Rather, when examining final outcomes, particularly long-term outcomes (Mumford, 2006), results consistently indicate that all three pathways are possible routes to achievement with no one pathway emerging as a dominant avenue to success. As such, CIP offers one glimpse into how equifinality can be embraced in the study and application of leadership. There is further hope, moreover, with organizations such as Gallup also recommending the principle be embraced more fully (Musser, 2019).

## How Can Equifinality Help Reduce Conflict?

At the outset of our discussion, we offered that the organizations we engage with most are increasing in diversity, a natural by-product of a changing nation and workforce. Hinted at in this discussion but not discussed expressly is greater tension—and even violence—in the workplace across lines that, on the surface, differentiate us. Such tension seems particularly taught when discussing leaders, who represent us in places we cannot attend ourselves. Candidly, given recent turmoil, it would be willfully naïve to suggest that we can wave our academic hands and simply make that tension go away.

However, despite such turmoil, we are optimistic in embracing the principle that a collective shift in openness of multiple pathways to success, in some cases centered on leadership, can take the edge out of perceived conflict. Consider a scenario where we view a given leader not as suggesting their way is superior but rather as using a pathway that is best for them. The sentiment here is that the style one leader uses does not inherently represent a challenging of the style another leader chooses. There is room for multiple pathways to the same outcome. Many roads lead to Rome, so to speak.

Admittedly, there is some gaudiness in the notion that calling for a shift in how leaders are simply thought of can have a substantive impact in how leaders and followers shift their thinking on what leadership looks like. Yet, we have seen it. Transformational leadership was born from the initially obscure work of James MacGregor Burns, who used it to study and think about political leaders. Bernard Bass was stuck on an airplane tarmac and, as the story goes, drafted an extended version of the theory on the literal back of a napkin. Transformational leadership not only took over the field of leadership, but in many ways saved it (Hunt, 1999). As scholars gave talks, consultants took notice and began developing and training leaders in the vein of charisma and transformational approaches (see, for example, Deloitte’s transformational leadership training services). Students were also trained in that environment, eventually seeing one primary way to think of leadership. From the back of a napkin to a generation of leadership researchers, it is certainly *possible* for a theoretical framework to shape a generation of leaders. We believe that this theoretical framework need not be limited to one style of leadership. And more, a broader acceptance of the principle of equifinality creates needed space for different types of leaders to have impact and shift our focus from seeing our differences as competition to accepting them as one approach among many.

## What Does Equifinality Mean for Developing Future Leaders?

Having introduced the notion of equifinality, and if the reader is convinced of the potential use of the premise, the emergent question becomes: How do we leverage this principle in developing and supporting leaders? Flowing from similar efforts that focus on a more holistic approach to developing leaders (e.g., Lindsay & Friesen, 2020), we offer that the answer is two-fold. First, embracing the principle of equifinality means shifting a mindset from one of identifying the best way to identifying one good way. Although more varied and interdisciplinary approaches are recommended by reports such as the National Leadership Education Research Agenda (e.g., Andenoro & Skendall, 2020; Lindsay & Friesen, 2020), an equifinality approach is often easier said than done. We are naturally inclined toward competition, be that against others or simply in seeking a solution to a problem. Embracing equifinality means breaking a few bad habits, a challenge particularly faced by adult learners.

Second, and related to the first, is an active seeking of alternative pathways. Should a viable approach to leading emerge, it is tempting to conclude this is the way it *should* be done by other leaders from that point on. However, when a mentee asks our advice on leading, it is imperative that we offer alternative pathways and begin to guide younger leaders into thinking about equifinality in their approach to leadership. Providing such guidance, however, means having pathways to offer. As such, we must be open to these pathways, cognitively tuck away and remember pathways as we witness them and express them when we are able. As a general technique, coaching emerging leaders to embrace a culture that embodies equifinality would help further elicit recognition and application of multiple leadership pathways. In addition, leaders who simply role-model an equifinality approach can further serve to guide young and emerging leaders toward embracing alternative paths of leading.

In line with the above is the potential for an adopted equifinality framework to expand options for leading to go beyond typical or traditional leadership structures. That is, in addition to stylistic approaches such as CIP, the equifinality principle could be used as a catalyst to embrace shared leadership structures such as dual or co-leadership (Hunter et al., 2017), or used to encourage more collaborative structures to tackle the complex problems of modern civilian and military organizations. Broadly, an equifinality-based approach to leading encourages decision makers in organizations to push past traditional boundaries and think about how a given situation might be leveraged to find a unique, but still viable, pathway to success.

## Concluding Comments

As we close, a few key points and caveats should be borne in mind. The first is subtle, noting that there are several nuances to embracing equifinality as a core concept in leadership. Namely, we do not suggest that all leadership approaches are equivalent in their ability to produce successful outcomes. Indeed, a laissez-fare approach to leading is inferior to either transformational or transactional approaches. An approach that embraces coercion as a power base will be less effective in the long run than an approach that embraces referent or expert power bases. There is such a thing as bad leadership, and we do not mean to equivocate poor performance with a stylistic difference.

Rather, we offer that there is utility in *being open to the potential* for equifinality. That is, when a leader engages in a style that differs from one’s own or from traditionally employed approaches, being open to that approach as a viable alternative pathway is a good starting point. A different approach need not inherently challenge our own style and approach to leading. That leader may simply be finding their own path and one that can allow them and their followers to find the same outcome as we are capable of.
